# Improving Oxidative Stress Through a Wheat Aleurone-Rich Diet: Are Short-Chain Fatty Acids Possible Mediators?

**DOI:** 10.3390/nu17203290

**Published:** 2025-10-20

**Authors:** Roberta Testa, Dominic Salamone, Angela A. Rivellese, Gabriele Riccardi, Marilena Vitale, Rosalba Giacco, Giuseppina Costabile

**Affiliations:** 1 Department of Clinical Medicine and Surgery, Federico II University, 80131 Naples, Italy; roberta.testa@unina.it (R.T.); rivelles@unina.it (A.A.R.); gabriele.riccardi@unina.it (G.R.); marilena.vitale@unina.it (M.V.); 2 Department of Agricultural Sciences, University of Naples “Federico II”, 80055 Portici, Italy; dominic.salamone@unina.it; 3 Institute of Food Sciences, National Research Council, 83100 Avellino, Italy; rgiacco@isa.cnr.it

**Keywords:** short chain fatty acids, wheat aleurone, whole-grain cereals, oxidative stress, 8-isoprostane

## Abstract

**Background/Objectives**: Dietary fibers from cereals promote the production of short-chain fatty acids (SCFA), which have been linked to improved glucose and lipid metabolism, reduced inflammation, and decreased oxidative stress. Wheat aleurone, a bran fraction enriched in fermentable fibers and bioactive compounds, may enhance SCFA production, but clinical evidence remains limited. This study investigated whether a wheat aleurone-rich diet, compared with a refined wheat diet, modulates circulating SCFA concentrations and their relationship with oxidative stress in individuals at elevated cardio-metabolic risk. **Methods**: In a randomized, cross-over trial, 23 adults with abdominal obesity and at least one additional metabolic syndrome feature consumed isoenergetic diets enriched with wheat aleurone or refined wheat for 8 weeks. Fasting and postprandial serum SCFA concentrations were measured over 3 h following standardized test meals using the gas chromatography method. Urinary 8-isoprostane excretion was assessed as a biomarker of oxidative stress using the ELISA method. SCFA values are reported as changes (increase/decrease) from fasting values, calculated by subtracting the fasting value from that of each time point. **Results**: Compared with refined wheat, the wheat aleurone diet significantly increased postprandial butyrate response (*p* = 0.005, time × meal interaction), with higher values observed at 150 min (*p* = 0.027) and 180 min (*p* = 0.001). The mean change in postprandial butyrate was also greater after the wheat aleurone diet (+0.95 ± 1.92 vs. −0.32 ± 2.01 µmol/L; *p* = 0.040). Importantly, butyrate at 180 min was inversely correlated with urinary 8-isoprostane (r = −0.618, *p* = 0.019). No significant differences were found for acetate or propionate. **Conclusions**: A wheat aleurone-rich diet enhances postprandial butyrate production and is associated with lower oxidative stress, suggesting a role of butyrate in mediating the antioxidant benefits of wheat aleurone in individuals with cardio-metabolic risk. This study is registered under ClinicalTrials.gov Identifier no. NCT02150356.

## 1. Introduction

In recent years, growing attention has focused on elucidating the specific contribution of cereal-derived dietary fibers to short-chain fatty acid (SCFA) production and their potential role in modulating lipid and glucose metabolism as well as oxidative stress [1,2]. Evidence from human studies indicates that the intake of cereal-derived fibers is associated with increased fecal and circulating concentrations of propionate and butyrate, both in healthy individuals and in populations at high cardiovascular risk [1,2,3]. Moreover, an interventional study reported that a diet enriched in whole grains led to increased serum propionate concentrations and this increase was associated with an improved postprandial insulin sensitivity in individuals at elevated cardiovascular risk [4]. In vitro and in vivo studies have elucidated potential mechanisms through which SCFAs regulate glucose and lipid metabolism at both the intestinal and systemic levels, as well as inflammation and oxidative stress [5,6,7,8,9,10]. In particular, butyrate may exert direct antioxidant effects by enhancing the activity of key antioxidant enzymes such as catalase (CAT), oxidoreductases, glutathione reductase, glutathione peroxidase, glutathione S-transferase, and superoxide dismutase-2 (SOD-2) [11].

Among the various components of bran, the aleurone layer contains a variety of bioactive compounds that could exert beneficial health effects. It represents the innermost part of the wheat bran and the outermost layer of the starchy endosperm, accounting approximately for 5–8% of the eight of the wheat kernels [12]. It is particularly rich in nutrients, containing substantial amounts of proteins (accounting for ~15% of the total wheat protein), minerals, phytates, B vitamins (mainly niacin and folates), lipid compounds such as plant sterols, lignans, and phenolic antioxidants—most notably 4-hydroxy-3-methoxycinnamic acid (ferulic acid)—as well as betaine [13,14]. The wheat aleurone is composed of a single layer of living cells whose walls consist predominantly of arabinoxylans (≈65%) and β-glucans (≈29%)—two highly fermentable fibers—together with a smaller fraction of cellulose [12]. Despite its richness in fermentable fibers and bioactive phytochemicals, evidence on the effects of wheat aleurone on the gut microbiota composition and its derived metabolites remains limited in humans [15].

Therefore, to assess the fermentative capacity of the aleurone fraction, in this study, we investigated the effect of a Wheat Aleurone diet—previously shown to reduce urinary 8-isoprostane, a well-established marker of oxidative stress [16]—on circulating SCFA levels in individuals at high cardiometabolic risk, compared with a Refined Wheat diet. Furthermore, we examined the potential associations between specific SCFA derived from aleurone fermentation and the reduction of oxidative stress, observed with the Wheat Aleurone diet [16].

## 2. Materials and Methods

### 2.1. Participants and Study Design

Twenty-three participants (11 males, 12 females) with abdominal obesity and at least one additional feature of metabolic syndrome, according to the National Cholesterol Education Program Adult Treatment Panel III (NCEP ATP III) criteria, were randomly assigned to follow two isoenergetic diets based on either wheat aleurone or refined wheat for 8 weeks, following a sequential cross-over design (Figure S1).

Full details of the study design and dietary interventions have been published previously [16]. Briefly, participants were instructed to maintain their habitual intake of meat, dairy products, eggs, fish, fruits, vegetables, and fats throughout the study. The only difference between the two diets was the replacement of cereal-based products with either aleurone-enriched or refined wheat equivalents. The aleurone-enriched products contained 8–15% aleurone in bread, 20% in pasta, and 8% in biscuits. All products were supplied by Barilla S.p.A. (Parma, Italy), except for the 15% aleurone bread, which was locally produced using industrially standardized aleurone (particle size < 100 µm). The refined wheat diet included corresponding products made from standard white wheat flour. Table 1 reports the chemical composition of the wheat aleurone-enriched and refined wheat products used in the study. Products containing the aleurone fraction showed higher levels of minerals (Mg, P, Fe), B-group vitamins, and particularly ferulic acid compared to the refined counterparts. Ferulic acid content was markedly increased in the aleurone-enriched products (401 mg/100 g in pasta, 181 mg/100 g in biscuits, and 225 mg/100 g in bread) versus the refined ones (150, 73, and 187 mg/100 g, respectively).

Aleurone supplementation increased cereal fiber intake by approximately 9 g/day, resulting in a cereal fiber intake of ~20 g/day (Table S1). To support compliance, participants were provided with sufficient quantities of study products for home consumption throughout the intervention period.

At baseline and at the end of each 8-week dietary intervention, clinical evaluations—including body weight, waist circumference, and blood pressure—were conducted, and fasting blood samples were collected for the assessment of biochemical markers. In addition, participants underwent a 3 h postprandial metabolic test following consumption of a standardized test meal that reflected the assigned diet. The standardized test meals (~1000 kcal; 14.5% protein, 38.8% fat, 50.6% carbohydrates) differed only in aleurone content (27 g vs. 0 g), total fiber (18.7 g vs. 13.1 g), and aleurone-derived fiber (5.64 g vs. 0 g), while fiber from other sources was kept constant.

Blood samples were collected in K2-EDTA tubes over the 3 h period for the measurement of short-chain fatty acids (SCFAs). Additionally, 24 h urine samples were collected to assess urinary 8-isoprostane levels as a marker of oxidative stress (Figure S1).

Dietary compliance was assessed through a 7-day food record completed by participants during the study period and by measuring fasting plasma concentrations of total alkylresorcinols (ARs) as previously described [16].

The study, registered on ClinicalTrials.gov (Identifier: NCT02150356), was conducted in accordance with the Declaration of Helsinki and was approved by the Ethics Committee of the University of Naples Federico II (Approval No. 209/13). Written informed consent was obtained from all participants.

### 2.2. Laboratory Methods

Urinary 8-isoprostane concentrations were measured using a sandwich enzyme-linked immunosorbent assay (ELISA) kit (Cayman Chemical, Ann Arbor, MI, USA) and read on a Triturus Analyser (Diagnostics Grifols, S.A., Barcelona, Spain).

Serum short-chain fatty acids (SCFAs)—acetic, propionic, and butyric acids—were extracted and quantified by gas chromatography coupled with a flame ionization detector (GC/FID) (Dani, Analitica Instruments S.p.A., Milan, Italy). The analysis was performed using a megabore column compatible with aqueous solvents, according to the method described by Rémésy and Demigné [18]. Serum samples were deproteinized by the addition of metaphosphoric acid; under these conditions, positively charged proteins acted as polycations and co-precipitated with the acid. The precipitated proteins were removed by centrifugation, and SCFAs were subsequently analyzed in the supernatant by GC/FID. Isovaleric acid was used as an internal standard. A stock solution containing acetic, propionic, and butyric acids at low, medium, and high concentrations was used to construct the calibration curve. A control sample with known SCFA concentrations was injected at both the beginning and the end of each analytical run to monitor accuracy and retention time stability. The intra-assay coefficients of variation were 2.2%, 2.0%, and 1.2%, and the inter-assay coefficients were 2.9%, 2.0%, and 1.8% for acetate, propionate, and butyrate, respectively.

### 2.3. Statistical Analysis

The sample size was calculated based on the primary outcome of the trial (improvement in postprandial insulin response) [16]. Using data from a previous study that compared inulin and glucose and reported differences in postprandial butyric acid response during the first 4 h after meal intake [19], assuming a mean treatment difference of 19% with a two-sided significance level of 0.05 and 80% power, the required sample size was 18 participants, accounting for a 10% dropout rate.

Data are expressed as Mean ± Standard Deviation unless otherwise stated. Shapiro–Wilk-test was performed to check if variables followed a normal distribution. Variables not normally distributed were analyzed after logarithmic transformation. SCFA values are reported as changes (increase/decrease) from fasting values, calculated by subtracting the fasting value from that of each time point. Overall postprandial responses were evaluated by two-way repeated measures ANOVA during the entire postprandial profile (180 min) to examine the effects of time, meal and the interaction time x meal. In detail, SCFA concentrations at time 30, 90, 150 and 180 min were included as levels of the within-subject “time” factor, and the Wheat Aleurone meal and Refined Wheat meal were included as levels of the within-subject “meal”. A generalized linear model (GLM) was used to evaluate differences between the two dietary treatments including the sequence treatment as covariate. Differences from baseline in each dietary treatment were evaluated by paired *t*-test. Bivariate associations were assessed between SCFA and urinary isoprostane concentration by Spearman’s correlation. A *p* value < 0.05 was considered significant. The statistical analysis was performed using SPSS software 27.0 (SPSS/PC, IBM, Armonk, NY, USA).

## 3. Results

Twenty-three participants (11 males, 12 females) completed the study (Figure S2). As previously described [16], compliance was high for both dietary interventions. Throughout both study periods, participants reported regular consumption of the recommended portions of bread, pasta, and biscuits enriched with aleurone or their refined wheat counterparts. In line with the study design, the Wheat Aleurone Diet provided a significantly higher fiber intake compared to the Refined Wheat Diet, with an estimated difference of approximately 9 g/day in both total and cereal fiber content (*p* < 0.001) (Table S1). Following both dietary interventions, as already published [16] no significant changes were observed in clinical and metabolic parameters while urinary 8-isoprostane levels were significantly lower after the Wheat Aleurone Diet compared to the Refined Wheat Diet (874 ± 346 vs. 1303 ± 874 ng/24 h, *p* = 0.035) (Table 1). Serum fasting concentrations of SCFAs (acetic, propionic, and butyric acid) did not differ significantly between the dietary interventions (Table 2).

### Postprandial SCFA Profile

**Figure 1:**
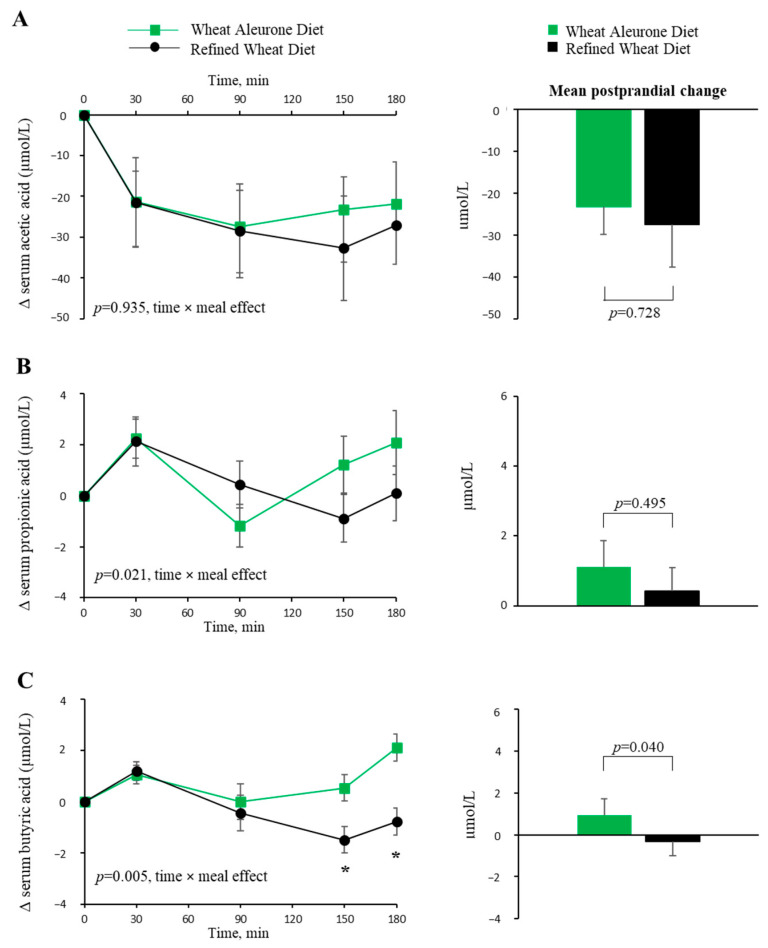
Postprandial changes from fasting levels (Δ) and mean postprandial changes in serum acetic (**A**), propionic (**B**), and butyric acid (**C**) concentrations following consumption of a wheat aleurone diet and a wheat refined diet. Green lines and bars are for “wheat aleurone diet”; Black lines and bars are for “refined wheat diet”. Data are expressed as Mean ± SEM; * *p* < 0.05, Comparison between Wheat Aleurone Diet vs. Refined Wheat Diet made by GLM-Univariate analysis adjusted for dietary treatments sequence.

No differences were observed in the postprandial acetic acid response (expressed as changes vs. fasting) (*p* = 0.935, time x meal effect) or in the mean postprandial change (*p* = 0.728) between the Wheat Aleurone Diet and the Refined Wheat Diet (Figure 1A).

The postprandial propionic acid response (expressed as changes vs. fasting) differed between the two diets (*p* = 0.021, time × meal effect) showing a more pronounced increase from 90 min after the Wheat Aleurone Diet compared to the Refined Wheat Diet. However, no significant differences were observed at individual time points along the postprandial curve, nor in the mean postprandial change of propionic acid (*p* = 0.495) (Figure 1B).

The postprandial butyric acid response (expressed as changes vs. fasting) differed between the two diets (*p* = 0.005, time × meal effect). In particular, butyrate levels began to rise 90 min after meal ingestion, reaching significantly higher concentrations at 150 min (*p* = 0.027) and 180 min (*p* = 0.001) following the Wheat Aleurone Diet compared to the Refined Wheat Diet. Moreover, the mean postprandial change in butyric acid was significantly greater after the Wheat Aleurone Diet than after the Refined Wheat Diet (0.95 ± 1.92 vs. −0.32 ± 2.01 µmol/L; *p* = 0.040) (Figure 1C).

**Figure 2:**
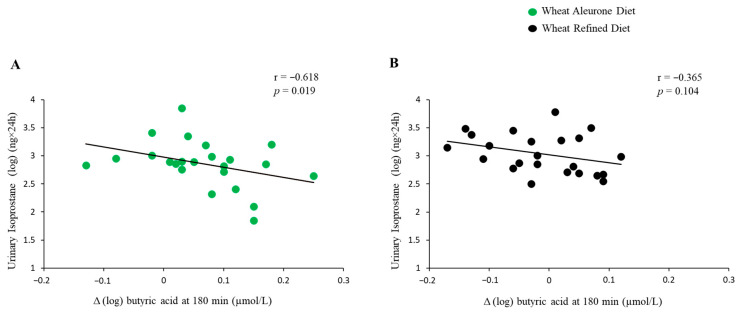
Correlations between postprandial changes from fasting levels (Δ) in butyric acid at 180 min and urinary isoprostane concentrations during the Wheat Aleurone Diet (**A**) and Refined Wheat Diet (**B**). Green circles are for “wheat aleurone diet”; black circles are for “refined wheat diet”.

Correlation analyses were conducted by analyzing each dietary period separately. The butyric acid postprandial response at the 180 min time point showed a significant inverse correlation with urinary 8-isoprostane levels after the Wheat Aleurone Diet (r = −0.618, *p* = 0.019), but not after the Refined Wheat Diet (r = −0.365, *p* = 0.104) (Figure 2). No statistically significant correlations were detected between urinary 8-isoprostane levels and either acetic or propionic acid response after both standard test meals.

## 4. Discussion

The most relevant finding of this study is that the wheat aleurone fraction was effectively fermented, leading to a significant increase in postprandial serum butyric acid concentrations. Notably, this rise in circulating butyric acid was inversely associated with urinary 8-isoprostane levels, a recognized biomarker of oxidative stress, and this relationship was evident only during the Wheat Aleurone Diet. These findings suggest that butyric acid may contribute to the modulation of oxidative stress, potentially accounting for the reduction in urinary 8-isoprostane levels previously reported in the intervention study [16].

Aleurone is a cereal bran fraction rich in bioactive compounds, including polyphenols, fibers, minerals, and vitamins, which may modulate the composition and metabolic activity of the gut microbiota. Among these components, arabinoxylans and β-glucans have been identified as key dietary fibers capable of stimulating SCFA production, particularly butyric acid. Some studies have demonstrated that foods rich in arabinoxylans can increase both serum and fecal concentrations of butyric acid [3,20]. Similarly, β-glucans have been shown to modulate gut microbiota composition, enhancing the abundance of SCFA-producing bacteria and thereby contributing to higher SCFA levels [21].

The elevated butyric acid concentrations observed following the Wheat Aleurone Diet may be attributed to the specific fiber content provided by the aleurone fraction (approximately 9 g). This contribution effectively increased total fiber intake in the Wheat Aleurone Diet and selectively enhanced the intake of fermentable fibers such as arabinoxylans and β-glucans, which are known to support butyrate production through microbial metabolism. To the best of our knowledge, only one human study has reported a modification in gut microbiota composition following four weeks of consumption of aleurone-enriched foods, showing an increase in Bifidobacterium and Lactobacillus, but no significant changes in fecal SCFA concentrations [15]. In vitro studies have shown that fermentation of aleurone-derived fibers increases the production of short-chain fatty acids (SCFAs), primarily butyric acid [22,23]. Another important aspect of our findings is the inverse relationship between postprandial butyric acid concentrations and oxidative stress, as assessed by urinary 8-isoprostane levels. Butyrate exerts important anti-inflammatory effects both at the intestinal and systemic level, and one of the mechanisms through which it may exert this function is by reducing oxidative stress [11,24]. However, acute supplementation of sodium butyrate in humans did not affect oxidative stress, as assessed by nitric oxide activity and glutathione peroxidase activity [25]. Isoprostanes are markers of lipid peroxidation and are prostaglandin-like compounds formed in vivo through non-enzymatic mechanisms involving the free radical-mediated peroxidation of arachidonic acid [26]. Butyrate may reduce lipid peroxidation by decreasing the production of free radicals. Both in vitro and in vivo studies have demonstrated that butyrate can act through multiple pathways to mitigate oxidative stress [11]. In particular, it can modulate the activity of antioxidant enzymes, protect mitochondrial function, and influence gene expression related to oxidative stress and inflammation [27]. Butyrate has been shown to increase the expression and activity of antioxidant enzymes such as CAT, oxidoreductases, glutathione reductase, glutathione peroxidase, glutathione S-transferase, and SOD-2, thereby neutralizing free radical production [28,29,30]. Oxidative stress can damage mitochondria, leading to impaired energy production and further oxidative injury [31]. Moreover, butyric acid can enhance the activity of adenosine monophosphate-activated protein kinase (AMPK) and improve mitochondrial function, thereby reducing the production of reactive oxygen species (ROS) [32]. Another potential mechanism involves the activation of the cyclooxygenase-2 (COX-2)/nuclear factor erythroid 2–related factor 2 (Nrf2)/heme oxygenase-1 (HO-1) pathway through the upregulation of COX-2, Nrf2, and HO-1 expression, along with the inhibition of nuclear factor kappa-light-chain-enhancer of activated B cells (NF-κB) expression, thereby reducing the inflammatory state [33,34].

A contribution to the reduction of oxidative stress, as observed in our previous study [16], may also be attributed to the high ferulic acid content of the wheat aleurone-based products used. Ferulic acid is a well-known antioxidant compound, acting as an effective scavenger of free radicals and an inhibitor of lipid peroxidation, thereby playing a key role in protecting against oxidative damage. However, we demonstrated that consumption of the aleurone-rich diet did not significantly alter the concentrations of total and individual urinary ferulic acid metabolites, suggesting that its bioavailability and metabolic conversion in vivo may be limited [16]. Nevertheless, we cannot exclude that even a modest increase in urinary ferulic acid metabolites could exert a beneficial effect on the oxidative status over the long term [16].

In our study, we did not observe a real increase in serum propionic acid levels following the Wheat Aleurone Diet unlike what we observed in a previous study, where 12-week wholegrain-based diet increased plasma propionate concentrations compared to a refined-cereal-based diet used as control, and this increase correlated with an improved insulin postprandial response in individuals with metabolic syndrome [4]. The discrepancy may be attributed to the different amounts of cereal fiber utilized in the two studies (28.9 g/day in the previous study and 20 g/day in the present one).

It is worth noting that fasting SCFA concentrations (Table 1) did not differ significantly between the two dietary interventions. This finding is consistent with previous evidence showing that fasting SCFA levels remain relatively stable and are mainly influenced by the previous evening meal rather than by long-term dietary intake [3]. In our previous eight-week intervention comparing a Mediterranean diet rich in fiber from multiple plant sources with a Western-style diet [35], we similarly observed no significant changes in fasting serum SCFA concentrations, whereas a marked increase in butyric acid concentrations was detected. This suggests that, even after prolonged dietary exposure, assessing intestinal fermentation in the postprandial phase is essential to capture the dynamic metabolic effects of fiber-rich foods. Accordingly, postprandial measurements provide a more sensitive reflection of colonic fermentation, which occurs several hours after ingestion.

Our study has both strengths and limitations. One of the main strengths is the high adherence of participants to both dietary interventions. This was confirmed by the measurement of alkylresorcinols, as already reported [16] which served as a reliable biomarker for the intake of aleurone-enriched cereals. Furthermore, the intervention with aleurone led to a significant 15% increase in plasma betaine levels, further supporting a good compliance [16].

These biomarkers provide robust evidence that participants effectively followed the dietary protocols, strengthening the validity of our findings. Another strength of the study is its crossover design, which allowed each participant to serve as their own control, minimizing inter-individual variability and increasing the statistical power to detect diet-related effects. However, the absence of a washout period may represent a limitation. Nonetheless, statistical analyses were adjusted for treatment sequence, thus accounting for potential carryover effects. Another limitation is the lack of gut microbiota composition data, which would have allowed us to explore microbiota-mediated differences between the two diets and to better explain the variations observed in SCFA profiles. In addition, the SCFA analysis was limited to serum concentrations measured during the first three hours after the test meal, which may have prevented the detection of statistically significant differences in the later postprandial period. In fact, it can be assumed that SCFA production continues to increase up to 6–8 h after the consumption of the aleurone-rich meal, as reported in previous postprandial kinetic studies showing a delayed rise in SCFA levels during the later phase of colonic fermentation. [3,8,18,36,37,38].

Moreover, a relatively high variability was observed in the relationship between postprandial butyric acid response at 180 min and urinary 8-isoprostane levels in the Wheat Aleurone Diet (Figure 2A). Nevertheless, the correlation coefficient (r = −0.618, *p* = 0.019) indicates a moderately strong inverse association, suggesting that higher postprandial butyric acid levels may be linked to lower oxidative stress. This dispersion may reflect inter-individual differences in SCFA production and metabolism that were not completely controlled for, even within the crossover design. Such variability may arise from differences in gut microbiota composition, colonic fermentation capacity, and SCFA absorption rates, all of which are known to influence systemic SCFA availability and related metabolic responses.

Finally, the relatively small sample size may have limited the statistical power to detect subtle associations between SCFA and oxidative stress markers. Therefore, further studies involving larger populations are warranted to confirm the antioxidant effects of aleurone and to better elucidate the potential mediating role of SCFA. Nevertheless, the present findings can be considered as preliminary evidence supporting a possible antioxidant role of aleurone through the modulation of SCFA production, particularly of butyric acid.

## 5. Conclusions

In conclusion, to the best of our knowledge, this is the first study in humans evaluating the effects of aleurone on the production of SCFA and on their potential role in the modulation of the oxidative stress. Our study has demonstrated that the inclusion of aleurone-enriched cereal products in the diet enhances the fermentative activity of the gut microbiota, as indicated by increased serum postprandial butyric acid levels. This increase can contribute to the improvement of oxidative stress.

## Figures and Tables

**Table 1:** Chemical composition of wheat aleurone products and refined wheat products used in the study.

	Wheat Aleurone Pasta	Wheat Refined Pasta	Wheat Aleurone Biscuits	Wheat Refined Biscuits	Wheat Aleurone Bread	Wheat Refined Bread
Energy (kcal/100 g)	356	359	432	436	271	269
Proteins (g/100 g)	14.76	12.01	8.84	7.93	10.72	10.73
Fats (g/100 g)	2.76	2.03	12.33	12.00	5.37	5.40
SFA (g/100 g)	0.52	0.50	3.15	3.15	0.9	0.9
MUFA (g/100 g)	0.57	0.40	7.47	7.40	3.47	3.50
PUFA (g/100 g)	1.66	1.10	1.72	1.50	1.00	1.00
Carbohydrates (g/100 g)	64.45	71.70	68.65	72.03	41.81	41.04
Sugars (g/100 g)	4.71	3.50	21.20	20.50	6.87	6.56
Total Fibers (g/100 g)	6.92	3.00	5.59	4.20	6.08	6.86
Sodium (mg/100 g)	4.7	5	183	187	538	535
Magnesium (mg/100 g)	154	75.00	73.9	29.2	72.6	68.1
Phosphorus (mg/100 g)	481	350	243	110	233	221
Iron (mg/100 g)	5.00	1.40	2.7	1.25	2.61	2.42
Vitamin B1(mg/100 g)	0.26	0.15	0.17	0.11	0.14	0.12
Vitamin B3 (mg/100 g)	1.72	0.90	1.7	0.24	1.09	0.915
Ferulic Acids (mg/100 g)	401	150	181	73	225	187
TEAC (mmol/100 g)	1.51	1.50	1.65	1.51	1.52	1.42

All analytical determinations were performed according to standard AOAC methods [17].

**Table 2:** Urinary isoprostanes and fasting serum short chain fatty acids of the participants at baseline and after the two 8-week dietary treatments.

	Baseline (*n* = 23)	Wheat Aleurone Diet (*n* = 23)	Refined Wheat Diet (*n* = 23)	*p* Value (Between Diets) ^a^
Urinary isoprostane (ng × 24 h)	1149 ± 636	874 ± 346 ^b^	1303 ± 874	0.035
Fasting Serum acetic acid (μmol/L)	210.48 ± 75.23	216.44 ± 61.77	218.93 ± 79.85	0.962
Fasting Serum propionic acid (μmol/L)	20.85 ± 5.57	20.45 ± 4.95	20.83 ± 6.79	0.821
Fasting Serum butyric acid (μmol/L)	12.63 ± 2.92	11.96 ± 2.58	13.41 ± 3.85	0.150

All values are mean ± SD; ^a^ Comparisons made by GLM-Univariate analysis adjusted for dietary treatments sequence; ^b^ *p* < 0.05. paired *t*-test vs. baseline.

## Data Availability

The data presented in this study are available on request from the corresponding author. (the data are not publicly available due to privacy and ethical restrictions).

## Supplementary Materials

The following supporting information can be downloaded at: https://www.mdpi.com/article/10.3390/nu17203290/s1, Figure S1: Study Design; Figure S2: Participant flow diagram; Table S1: Energy intake and nutrient composition of the diets at baseline and after the two 8-week dietary treatments.
