# Supplementary material for: Improving Oxidative Stress Through a Wheat Aleurone-Rich Diet: Are Short-Chain Fatty Acids Possible Mediators?
Source: Nutrients. 2025 Oct 20;17(20):3290. doi: 10.3390/nu17203290 (PMC12567320; doi:10.3390/nu17203290)
Supplement: Supplementary file 1 [file nutrients-17-03290-s001.zip › Table S1..docx]

**Table S1.** Energy intake and nutrient composition of the diets at baseline and after the two 8-week dietary treatments.

|  | **Baseline**  (n=23) | **Wheat Aleurone Diet**  (n= 23) | **Refined Wheat Diet**  (n= 23) | ***p value***  *(between diets)^a^* |
| --- | --- | --- | --- | --- |
| Energy (kcal) | 1870±444 | 1832±351 | 1802±449 | 0.933 |
| Protein (%) | 17±2.0 | 19±1.7^b^ | 19±1.9^b^ | 0.112 |
| CHO (%) | 50±3.4 | 51±3.7 | 50±4.2 | 0.854 |
| Fat (%) | 33±2.8 | 30±4.0^b^ | 31±5.0^b^ | 0.481 |
| SFA (%) | 8.8±1.6 | 6.8±1.4 | 7.7±2.3 | 0.347 |
| MUFA (%) | 12.9±1.6 | 15.7±1.6^b^ | 13.9±1.6 | 0.002 |
| PUFA (%) | 3.7±0.7 | 4.5±1.0^b^ | 3.7±1.0 | 0.015 |
| Cholesterol (mg/day) | 209±75 | 141±34^b^ | 161±64^b^ | 0.901 |
| Total Fiber (g/day) | 21±5 | 34±7^b^ | 25±8 | 0.001 |
| Cereal Fiber (g/day) | 11±2 | 20±4 ^b^ | 11±3 | 0.001 |
| Glycemic Index | 58±3 | 56±6 | 57±4 | 0.813 |

All values are mean ± SD.

^a^Comparisons made by GLM- Univariate analysis adjusted for dietary treatments sequence.

^b^p<0.05 *vs.* Baseline (t-Test).
