# Supplementary material for: Improving Oxidative Stress Through a Wheat Aleurone-Rich Diet: Are Short-Chain Fatty Acids Possible Mediators?
Source: Nutrients. 2025 Oct 20;17(20):3290. doi: 10.3390/nu17203290 (PMC12567320; doi:10.3390/nu17203290)
Supplement: Supplementary file 1 [file nutrients-17-03290-s001.zip › Figure S2..doc]

**Figure S2: Flow diagram**

**Allocation**

**Analyzed**

**n=23**

**Enrollment**

Received Wheat Aleurone Diet intervention (n=11)

Discontinued intervention (job reasons, family reasons, unwilling to perform

post-intervention tests)

(n=4)

Received Refined Wheat Diet intervention (n=12)

Discontinued intervention (job reasons, family reasons, unwilling to perform

post-intervention tests)

(n=3)

Assessed for eligibility (n=70)

Excluded (n= 40)

♦  Not meeting inclusion criteria (n=25)

♦  Declined to participate (n=15)

Allocated to Refined Wheat Diet intervention (n=15)

• Received allocated intervention (n=15)

Allocated to Wheat Aleurone Diet intervention (n=15)

• Received allocated intervention (n=15)

Randomized (n=30)
